# Genetic Insights into Azoospermia and Severe Oligozoospermia: Discovering Seven SNPs through GWAS and In Silico Analysis

**DOI:** 10.3390/cimb46070389

**Published:** 2024-06-27

**Authors:** Alexia Chatziparasidou, Maria-Anna Kyrgiafini, Theologia Sarafidou, Katerina A. Moutou, Zissis Mamuris

**Affiliations:** 1 Laboratory of Genetics, Comparative and Evolutionary Biology, Department of Biochemistry and Biotechnology, University of Thessaly, Viopolis, Mezourlo, 41500 Larissa, Greece; 2 Embryolab IVF Unit, St. 173-175 Ethnikis Antistaseos, Kalamaria, 55134 Thessaloniki, Greece

**Keywords:** male infertility, genome-wide association study (GWAS), single nucleotide polymorphism (SNP), long intergenic non-coding RNAs (lincRNAs)

## Abstract

Azoospermia and severe oligozoospermia represent the most extreme forms of male infertility. Despite their prevalence, the genetic foundations of these conditions are not well understood, with only a limited number of genetic factors identified so far. This study aimed to identify single-nucleotide polymorphisms (SNPs) linked to both azoospermia and severe oligozoospermia. We conducted a genome-wide association study (GWAS) involving 280 Greek males with normal semen parameters and 85 Greek males diagnosed with either azoospermia or severe oligozoospermia. Following rigorous quality control measures, our analysis identified seven SNPs associated with azoospermia/severe oligozoospermia. An in silico functional annotation was subsequently used to further investigate their role. These SNPs, found in regions not previously associated with male reproductive disorders, suggest novel genetic pathways that may contribute to these forms of infertility and pave the way for future studies. Additionally, this study sheds light on the significant role of noncoding RNAs in the pathogenesis of male infertility, with three of the identified SNPs situated in long intergenic non-coding RNAs (lincRNAs). Our findings highlight the intricate genetic landscape of azoospermia and severe oligozoospermia, underlining the necessity for more detailed studies to fully grasp the underlying mechanisms and their potential for informing diagnostic and therapeutic strategies.

## 1. Introduction

Male infertility constitutes a significant global health concern, affecting approximately 7% of the male population [1], with recent studies indicating a progressive increase in incidence over the past decades [2]. This growing trend underscores the profound impact of male infertility not only affecting couples’ reproductive aspirations but also contributing to significant social and psychological burdens [3]. Within the spectrum of male reproductive disorders, azoospermia, and severe oligozoospermia are among the most severe forms. Azoospermia is defined by the total absence of sperm in the ejaculate and is identified in 15% of infertile men [4], while severe oligozoospermia is characterized by markedly low sperm concentrations (less than 5 million sperm/mL) [5]. These conditions not only reflect severe impairments in spermatogenesis but also raise complex diagnostic and therapeutic challenges [4,6]. The causes of azoospermia and severe oligozoospermia are multifaceted, involving a complex interplay of genetic, environmental, and lifestyle factors, making their management particularly challenging [7]. Notably, azoospermia is associated with a heightened risk, approximately 25%, of carrying genetic abnormalities [1]. Among these, Y chromosome microdeletions are recognized as significant causes of severe oligozoospermia and azoospermia [8]. However, the role of single nucleotide polymorphisms (SNPs) in male infertility further illustrates the genetic complexity underlying these conditions [1]. 

In recent years, technological advancements have led to an increase in the identification of SNPs associated with male infertility through next-generation sequencing [9], but despite these advancements, our understanding of the molecular mechanisms underlying male infertility remains incomplete, resulting in idiopathic infertility still being a common diagnosis [7,10]. Moreover, the predictive value of candidate genetic markers is not consistent across all cases, which adds complexity to the diagnosis and management of male infertility [10]. This complexity highlights the need for more in-depth research to identify SNPs associated with male infertility in different populations and specific subtypes. Furthermore, assisted reproductive technologies (ARTs), which bypass natural selection in sperm, potentially increase the risk of transmitting genetic abnormalities to subsequent generations [11,12]. Therefore, it is imperative to deepen our understanding of the critical factors that influence sperm development. This will not only help reduce the genetic risks associated with ARTs but also improve the early detection and diagnosis of infertility through more reliable genetic markers and SNPs.

From this perspective, genome-wide association studies (GWASs) emerge as pivotal tools for the investigation of male infertility. Therefore, our study aimed to perform a GWAS to identify SNPs associated with azoospermia and severe oligozoospermia. Furthermore, we conducted in silico functional annotation to decipher the biological roles of the implicated genes and the potential impact of the identified SNPs. Specifically, our GWAS included 365 men, dividing them into groups based on spermiogram parameters: the control group included normozoospermic men, while the case group included participants with azoospermia or severe oligozoospermia. We also examined the genes harboring associated variants and the SNPs using various databases and bioinformatics tools, aiming to gain a complete understanding of their functionality and their contributions to male fertility. This approach not only allows us to pinpoint genetic factors associated with male infertility but also to explore the broader biological implications of these genetic markers.

## 2. Materials and Methods

### 2.1. Study Participants

This study included 365 participants who were all Greek males. All men provided their written informed consent to join this study. After completing a questionnaire to collect information about height (m), weight (kg), age, clinical history, medication, healthy habits, etc., each participant donated a blood sample which was used for genomic DNA extraction and ejaculate for semen analysis. 

Sperm samples were collected through masturbation, following a minimum abstinence period of two to three days. The processing of sperm and semen analysis followed the guidelines outlined in the fifth edition (2010) of the World Health Organization (WHO) [13]. Specifically, parameters such as semen volume, sperm count, motility, morphology, etc. were evaluated. Cell vision counting slides (Tek-Event) were used for cell counting, and Nikon Eclipse TS100, E200, and Ts2 microscopes were employed for observation. To elaborate, the samples underwent classification using the seminogram results and reference values stipulated in the WHO guidelines. Thus, the control group consisted of 280 samples with normal semen parameters or normozoospermia (sperm count > 15 × 10^6^ mL^−1^, total sperm count > 39 × 10^6^, total motility > 40% motile sperm, progressive motility > 32% (Grade a + b) motile sperm, and sperm with normal morphology > 4%), and the case group consisted of 85 samples with azoospermia or severe oligozoospermia. Samples were characterized as azoospermic or severe oligozoospermic after at least two semen analyses (seminograms) performed at 2–4-week intervals, following 3–5 days of sexual abstinence, to ensure accuracy and consistency of the results. The demographic characteristics and healthy habits of these men are presented in Table 1. Although azoospermic and severe oligozoospermic individuals were both included in the case group, the demographic data are presented separately for each condition in Table 1. The *p*-values indicate that there are no statistically significant differences in factors that could affect fertility or the validity of the results between the studied groups (e.g., alcohol consumption, age, etc.).

It should be noted that semen and blood samples were collected in cooperation with the “Embryolab Fertility Clinic” (Thessaloniki, Greece) for the research program “Spermogene” (Grant number Τ1ΕΔΚ-02787). Except for patients in the IVF unit, many of the participants were volunteers. Consequently, there is a difference in the numbers of diseased and control groups because we did not have prior information about the men’s fertility status. We also included all the samples collected in this study to increase the statistical power of our study. This study was also approved by the Ethics Committee of the University of Thessaly and was carried out in accordance with the guidelines of The Declaration of Helsinki.

### 2.2. Genotyping

Genomic DNA was extracted from blood samples using the PureLink Genomic DNA Mini Kit (Invitrogen, Waltham, MA, USA-Catalog number: K182002), following the manufacturer’s protocol. Briefly, 200 µL of whole blood was processed for each sample to ensure complete lysis of blood cells for efficient DNA release. Subsequently, the lysate underwent a column-based purification process, with DNA binding to a silica membrane in the presence of a chaotropic salt. Impurities were eliminated through a series of washing steps, resulting in the isolation of purified genomic DNA. The purified DNA was then eluted in a low-salt buffer optimized to preserve DNA quality and stability. To assess the concentration and purity of the extracted DNA, spectrophotometric measurements were carried out using a Qubit 2.0 fluorometer in conjunction with the Qubit dsDNA BR Assay Kit (Invitrogen, Waltham, MA, USA-Catalog number: Q32850). Furthermore, the integrity of the DNA was confirmed through agarose gel electrophoresis.

Once the preparation was completed, the purified DNA samples were transported to the Erasmus MC Human Genomics Facility (HuGe-F, University Medical Centre Rotterdam, The Netherlands) for genotyping. The genotyping was carried out using the Illumina Infinium^®^ Global Screening Array, a high-throughput genotyping platform capable of simultaneously genotyping more than 700,000 SNPs. These SNPs cover a wide range of genome-wide markers and variants that are relevant for pharmacogenomics and complex disease research. Additionally, the array utilizes Illumina’s Infinium assay, which combines whole-genome amplification, hybridization, extension, and staining steps to detect SNP alleles. This technology enables efficient and accurate genotyping of a large number of SNPs in a single assay.

### 2.3. Quality Control and Statistical Analysis

Genotype data obtained from the Illumina Infinium^®^ Global Screening Array were subjected to quality control processes using the PLINK software v1.07 [14]. To ensure the reliability and accuracy of our genomic data, we implemented stringent quality control criteria. Specifically, we excluded from further analysis samples with low call rates (<90%), SNPs with low call rates (<90%), deviation from the Hardy–Weinberg equilibrium (*p*-value < 0.001), and with a minor allele frequency (MAF) less than 3%. Furthermore, we applied exclusion criteria based on heterozygosity and relatedness tests and conducted SNP pruning to remove SNPs in linkage disequilibrium.

Then we used PLINK software v1.07 [14] to perform an association analysis, with the primary goal of investigating the relationship between SNP genotypes and the specific phenotypic trait of interest, which in this case was male infertility. To assess the significance of these associations, we employed Pearson’s chi-square test with a stringent threshold of *p*-value < 10^−5^. This allowed us to identify statistically significant SNP-phenotype associations. For visualization of our findings, we created Manhattan plots and quantile–quantile (Q–Q) plots using the *qqman* package version 0.1.9 [15].

### 2.4. In Silico ANALYSIS

In this study, we also focused on exploring the regulatory roles of SNPs identified as being significantly associated with male infertility after the association analysis. To enhance our understanding of these SNPs, we utilized various tools, including SNPnexus [16], the Genotype-Tissue Expression (GTEx) Portal [17], the 1000 Genomes Project database [18], RegulomeDB 2.2. [19], 3DSNP 2.0 [20], and miRNASNP-v3 databases [21]. These resources provided critical insights into regulatory elements, population genetics, and the potential functional impact of the identified SNPs. Specifically, SNPnexus [16] is particularly useful for annotating genetic variations and extracting significant insights from large genomic datasets, while the GTEx Portal [17] contains data on expression quantitative trait loci (eQTL) across various tissues, enhancing our understanding of gene expression patterns. Furthermore, RegulomeDB 2.2. [19] and 3DSNP 2.0 databases [20] were used to assess the functionality of SNPs. RegulomeDB 2.2. [19] classifies SNPs based on their regulatory potential and assigns them ranks from 1 to 7 to indicate the likelihood of regulatory impact. Similarly, 3DSNP 2.0 [20] offers a comprehensive view of the 3D genomic interactions, enhancer and promoter states, and potential impacts on transcription factor bindings and sequence motifs, using a scoring system to assess SNPs’ functionality. Finally, the miRNASNP-v3 database [21] was used to explore how SNPs might influence miRNA binding to mRNAs, potentially affecting gene regulation.

It should also be noted that all annotations performed were based on the Ensembl database [22] and the GRCh38 human reference genome, ensuring a comprehensive and up-to-date analysis framework.

## 3. Results

To elucidate the genetic underpinnings of male infertility, our study employed a genotyping approach, analyzing approximately 756,000 SNPs across 280 control subjects (men exhibiting normozoospermia) and 85 case subjects (men diagnosed with azoospermia or severe oligozoospermia). After applying strict quality control criteria, such as excluding samples with missing genotypes and SNPs with a very low MAF, etc., 214 controls, 72 cases, and approximately 236,000 SNPs were retained for subsequent association analysis utilizing the chi-square test.

**Figure 1:**
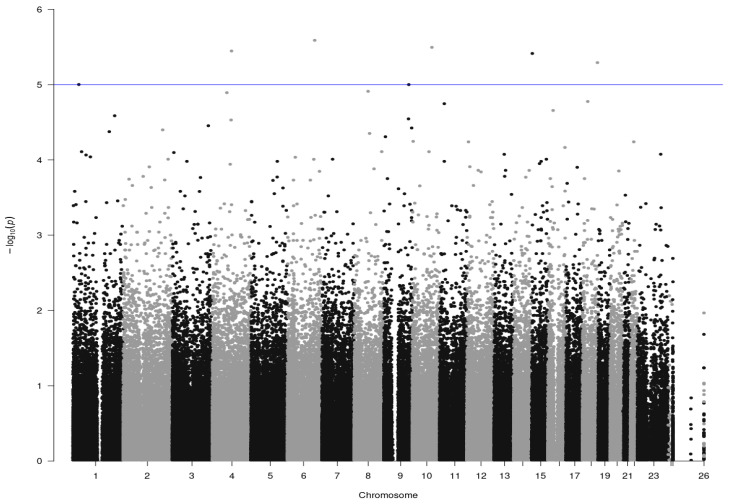
Manhattan plot of genome-wide association study data shows SNPs associated with azoospermia and/or severe oligozoospermia. The x-axis represents the genomic coordinates of SNPs on respective chromosomes, while the y-axis represents the significance level on a −log10 scale. The suggestive significance threshold is indicated by the blue horizontal line (*p*-value = 10^−5^).

The GWAS identified seven SNPs that showed a significant association with azoospermia and/or severe oligozoospermia, exceeding the established threshold for suggestive significance (*p*-value < 10^−5^). The results are presented in the Manhattan plot (Figure 1), which elucidates the genomic distribution of SNPs relative to their significance levels. Furthermore, the quantile–quantile (Q–Q) plot (Figure S1) is also provided, comparing the observed distribution of *p*-values against the expected distribution under the null hypothesis, as an indicator of the validity of the GWAS findings. 

As shown in Table 2 and Figure 1, the SNPs significantly associated with azoospermia/severe oligozoospermia are dispersed across various chromosomes, each correlated with an elevated risk of the condition being studied. It is also important to note that these SNPs have high odds ratios, indicating a strong association with azoospermia/severe oligozoospermia (Table 2). 

To elucidate the functional implications of statistically significant SNPs in the context of male infertility, a thorough annotation and in silico analysis were conducted using various databases, as previously explained. Firstly, population genetic data and allele frequency details across five distinct populations were obtained from the SNPnexus [16] and the 1000 Genomes Project [18] databases. As shown in Table 3, a notable finding is that two specific SNPs (rs75614542 and rs11572106) exhibited exceptionally low minor allele frequencies, each falling below the 0.05 threshold. Furthermore, a comparative analysis revealed that the allele frequencies in the case group (men with azoospermia/severe oligozoospermia) were consistently higher than those observed in the European population, with the most significant disparities observed for rs75614542 (a fivefold increase) and rs61712011 (a fourfold increase).

To deepen our understanding of the functional implications of the identified SNPs in azoospermia/severe oligozoospermia, we conducted a more extensive analysis using various databases and tools for comprehensive functional characterization, as previously explained. Table 4 shows that two of the significant SNPs (rs873041 and rs72963110) are located in intergenic regions, close to long intergenic non-coding RNAs (lincRNAs). Moreover, our analysis revealed three SNPs (rs77534195, rs61712011, and rs11572106) within intronic regions, one SNP (rs75614542) located in a 3′ untranslated region (UTR), and another SNP (rs17182744) mapped within a lincRNA region. It is noteworthy to mention that none of the identified SNPs were associated with expression quantitative trait loci (eQTL), according to the GTEx Portal [17]. In terms of their potential functional impact, three SNPs (rs873041, rs75614542, and rs11572106) were highly scored by RegulomeDB 2.2. [19], and one (rs17182744) was notable for its high 3DSNP 2.0 [20] score, indicating a strong likelihood of regulatory function.

Finally, the miRNASNP-v3 database [21] was employed to determine the impact of significant SNPs on mRNA-microRNA (miRNA) interactions. More specifically, an SNP located in the 3′ UTR region of the *FBXW2* gene generated new binding sites for four distinct miRNAs while concurrently causing the loss of a binding site for another miRNA. These findings are detailed in Table 5.

## 4. Discussion

Azoospermia and severe oligozoospermia are significant forms of male infertility. Genome-wide association studies (GWASs) have the potential to unravel the complex interplay of genes involved in spermatogenesis and testicular function, while also providing valuable markers for the enhancement of the diagnosis of male infertility. In this study, we conducted a GWAS involving 365 men, categorized into two groups: a control group with normozoospermia and a case group characterized by azoospermia or severe oligozoospermia. Our GWAS identified seven significant SNPs associated with azoospermia and severe oligozoospermia, showing markedly different allele frequencies between fertile and infertile men. Through extensive analysis using various databases and bioinformatics tools, we aimed to elucidate the functionality and impact of genes and SNPs associated with male fertility, offering insights into the genetic factors contributing to this condition.

Notably, the SNPs identified in this study are novel in the context of male infertility and have not been linked to any other pathological conditions previously. It should also be noted that the reported SNPs have not been associated with the progression of other diseases, even non-reproductive ones. Thus, there is a need for further studies on their role. However, the associated genes where these significant SNPs are located present intriguing possibilities for understanding their roles in male reproductive health, especially in azoospermia and severe oligozoospermia. 

More specifically, one SNP (rs77534195) was found in an intronic region of *GRID2*. *GRID2* encodes the Glutamate Ionotropic Receptor Delta Type Subunit 2, also known as GluD2, which is a part of the ionotropic glutamate receptors family [23]. These receptors are crucial for synaptic transmission in the brain and also play a significant role in synaptogenesis, synaptic plasticity, and motor coordination [24]. However, data from the Human Protein Atlas (www.proteinatlas.org, accessed on 20 April 2024) [25] indicate that *GRID2* expression is not limited to the brain, as it is also highly expressed in the testis. Specifically, *GRID2* is expressed in early spermatids, and its expression is even higher in the late spermatids stage. This dual high expression in both neurological and reproductive tissues suggests a potential, albeit less understood, role in testicular function and possibly in processes related to spermatogenesis or sperm development, opening the road for future studies.

Another SNP was also identified in the 3′ UTR region of the *FBXW2* gene, which encodes for a protein that is part of the F-box protein family. This family is characterized by an F-box motif and multiple WD-40 repeats, playing a vital role in cellular processes, primarily through ubiquitin-mediated degradation of cellular regulatory proteins [26]. The *FBXW2* gene exhibits low tissue specificity and is detected across various tissues, indicating its fundamental role in cellular processes. Studies also show that it acts as a tumor suppressor in several cancer types [27,28,29]. Furthermore, although this gene has not been previously linked to male infertility, another family member, *FBXW7*, has been shown to negatively regulate spermatogonia stem cell (SSC) self-renewal, highlighting the importance of F-box proteins in the self-renewal and differentiation of SSCs crucial for spermatogenesis [30]. The functional diversity of F-box proteins within SCF ubiquitin–ligase complexes further emphasizes their significant role in cellular regulation, affecting processes like cell cycle transitions and transcription regulation, which is essential for reproductive cell development and function [31]. Finally, the Human Protein Atlas [25] data reveal high *FBXW2* expression in the testis, particularly in late spermatids, suggesting a potential role of this gene in testicular function and spermatogenesis.

An SNP associated with azoospermia and severe oligozoospermia was also identified in an intronic region of the *CYP2C8* gene. CYP2C8 is a member of the cytochrome P450 family, which plays a role in metabolizing xenobiotics and polyunsaturated fatty acids [32]. Specifically, research has shown that CYP2C8 is involved in metabolizing more than 60 clinical drugs [33]. While there may not be specific studies directly linking cytochrome P450 enzymes to spermatogenesis, sperm function, or male infertility, there are indirect associations through their involvement in hormone metabolism and vitamin D regulation, both of which are crucial for male reproductive functions. For example, cytochrome P450 enzymes are involved in the metabolism of vitamin D, and it has been observed that sufficient levels of vitamin D are positively correlated with sperm motility and overall reproductive health [34]. Furthermore, cytochrome P450 enzymes are involved in cholesterol metabolism, which is essential for spermatogenesis. Cholesterol is a key component of cell membranes and serves as a precursor for steroid hormone biosynthesis, which is vital for the development and function of male reproductive tissues [35].

Furthermore, rs61712011 was found in an intronic region of the *SLC2A12* gene. The *SLC2A12* gene encodes GLUT12, a member of class III glucose transporters, which are responsible for transporting glucose and other substances across cellular membranes [36]. Although *SLC2A12* has been associated with certain types of cancer [37,38], specific details regarding its exact function, biochemical properties, and significance in human physiology are not as well-documented as other members of the SLC2 family [39]. Generally, glucose transporters encoded by *SLC2* genes play vital roles in metabolic processes and energy balance in various tissues [40]. While the available literature does not explicitly provide direct associations with spermatogenesis or male infertility, the importance of energy metabolism in reproductive processes suggests potential implications. Thus, further research could help elucidate the role of *SLC2A12* in testicular function and male reproductive health.

The present study also highlights the significant role of lncRNAs in male infertility, which is well established [41]. Specifically, three significant SNPs were identified in lincRNA regions or in close proximity to them. Although there are no studies about the function of *AC090457.1*, *SNHG14* is a well-studied lncRNA that has been extensively researched in various types of cancers, including gynecological cancers [42,43], colorectal cancer [44], hepatocellular carcinoma (HCC) [45], etc. Beyond its involvement in cancer, *SNHG14* has also been implicated in the genetic disorder Prader–Willi syndrome (PWS) [42]. However, its role in male infertility has not yet been explored. Finally, *LINC01756* is an understudied lincRNA, but it is also highly expressed in the testis, suggesting a potential regulatory role. Notably, two of the SNPs identified in lincRNA regions or close to them, also had a high RegulomeDB rank or 3DSNPscore, indicating their regulatory role. Furthermore, this study indicates the importance of miRNAs, as SNPs associated with azoospermia and severe oligozoospermia were found to either create or disrupt miRNA binding sites, potentially influencing gene regulation.

Regarding the strengths and limitations of the present study, one notable strength lies in the strict quality control criteria we employed. By carefully excluding SNPs and samples that could compromise the integrity of our findings, we reduced the risks of false positives and negatives. This rigorous approach, as described earlier, ensures the high quality of our results. Additionally, the utilization of sophisticated bioinformatics tools and comprehensive databases for data analysis has allowed for a precise interpretation of significant associations, thereby reinforcing the reliability of our conclusions. Finally, another strength is the genetic homogeneity of our sample, as all the volunteers completed a questionnaire about their ancestry. This further enhances the validity of our study by minimizing confounding variables associated with genetic diversity. However, this study also has some limitations. A primary constraint is the relatively small sample size, as only around 300 individuals remained after stringent quality control measures were applied. This limitation underscores the need for further, larger-scale genetic studies to fully evaluate the implications of the identified SNPs in azoospermia and severe oligozoospermia. Moreover, our study did not identify any SNPs meeting the conventional threshold for genome-wide significance (*p*-value < 5 × 10^−8^). Instead, we used a less strict cutoff, which can still provide reliable results, but interpretation should be executedwith caution.

In conclusion, this GWAS aimed to identify genetic variants associated with azoospermia and severe oligozoospermia in a cohort of Greek males. We discovered seven SNPs with notable associations, suggesting a nuanced genetic contribution to these conditions. Furthermore, we conducted an in silico analysis to explore their functional implications. Some of the genes hosting these significant SNPs have been implicated in biological processes not previously linked to male infertility, demonstrating the complex genetic architecture underlying these conditions. This study also highlights the significant role of noncoding RNAs in male infertility, as three out of the seven SNPs were found in lincRNAs or in close proximity to them. These findings open up new avenues for further research into the genetic factors contributing to male infertility, providing valuable insights for understanding, preventing, and managing these reproductive challenges.

## Figures and Tables

**Table 1:** Demographic characteristics and health habits of men selected to participate in this study. *p*-values (*p* > 0.05) indicate no statistically significant differences in demographic characteristics between the studied groups.

	Normozoospermic (*n* = 280)	Azoospermic (*n* = 43)	Severe Oligozoospermic (*n* = 42)	*p*-Value
**Body Mass Index—BMI (m/kg^2^)** **mean, SD**	17.9–40.4 25.9 (3.7)	21.5–47.6 28.4 (5.1)	24.1–37.3 27.7 (3.4)	0.905
**Age (years)** **mean, SD**	21–48 38.7 (8.3)	32–55 39.8 (5.5)	24–58 40.5 (7.8)	0.875
**Smoking (Yes/No)**	No, 56% Yes, 44%	No, 48.5% Yes, 51.5%	No, 50% Yes, 50%	0.665
**Alcohol Consumption (<2 drinks/week, 2 drinks/week, >2 drinks/week)**	<2 drinks/week, 58.8% 2 drinks/week, 20.6% >2 drinks/week, 20.6%	<2 drinks/week, 63.6% 2 drinks/week, 21.2% >2 drinks/week, 15.12%	<2 drinks/week, 58.3% 2 drinks/week, 25% >2 drinks/week, 16.7%	0.984

**Table 2:** Summary of the association results for azoospermia and/or severe oligozoospermia. Significant SNPs and their genomic position are presented; Chr, chromosome; Ref/Alt, reference/altered; OR, odds ratio.

Chr	SNP	Position	Ref/Alt Allele	Frequency Cases	Frequency Controls	*p*-Value	OR
1	rs873041	29,718,378	C/T	0.13190	0.032710	9.969 × 10^−6^	4.4950
4	rs77534195	94,718,164	C/A	0.11810	0.023360	3.573 × 10^−6^	5.5950
6	rs61712011	134,363,477	A/C	0.09722	0.014020	2.580 × 10^−6^	7.5740
9	rs75614542	123,521,842	A/C	0.09028	0.014020	9.996 × 10^−6^	6.9800
10	rs11572106	96,817,479	A/G	0.08333	0.009346	3.199 × 10^−6^	9.6360
15	rs17182744	25,279,455	C/T	0.11810	0.023470	3.856 × 10^−6^	5.5690
18	rs72963110	73,693,542	G/A	0.09028	0.012140	5.100 × 10^−6^	8.0780

**Table 3:** Allele frequencies in five populations for the SNPs found to be associated with azoospermia and/or severe oligozoospermia; Ref/Alt, reference/altered; Freq, frequency; MAF, minor allele frequency; EAS, East Asian; AMR, American; AFR, African; EUR, European; SAS, South Asian.

SNP	Ref/Alt Allele	Freq Cases	MAF	EAS	AMR	AFR	EUR	SAS
rs873041	C/T	0.13190	0.29	0.188	0.092	0.236	0.061	0.134
rs77534195	C/A	0.11810	0.17	0.098	0.027	0.022	0.081	0.101
rs61712011	A/C	0.09722	0.43	0.021	0.053	0.327	0.024	0.043
rs75614542	A/C	0.09028	0.04	0.000	0.009	0.000	0.017	0.007
rs11572106	A/G	0.08333	0.04	0.000	0.017	0.002	0.023	0.018
rs17182744	C/T	0.11810	0.06	0.000	0.016	0.016	0.034	0.018
rs72963110	G/A	0.09028	0.08	0.000	0.016	0.002	0.031	0.003

**Table 4:** Annotation and functional characterization of the SNPs found to be associated with azoospermia and/or severe oligozoospermia according to GTex portal, RegulomeDB 2.2., and 3DSNP 2.0.

SNP	Closest Gene	SNP-Gene Distance	Annotation	eQTL	RegulomeDB 2.2.	3DSNP Score
rs873041	*LINC01756* (lincRNA)	41,752 bp	Intergenic	No	Rank = 2b, Score = 0.61652	1.88
rs77534195	*GRID2*	0 bp	Intronic	No	Rank = 7, Score = 0.18412	2.07
rs61712011	*SLC2A12*	0 bp	Intronic	No	Rank = 7, Score = 0.51392	1.81
rs75614542	*FBXW2*	0 bp	3′ UTR	No	Rank = 1f, Score = 0.55324	6.63
rs11572106	*CYP2C8*	0 bp	Intronic	No	Rank = 1f, Score = 0.907	1.22
rs17182744	*SNHG14* (lincRNA)	0 bp	Non-coding	No	Rank = 7, Score = 0.18412	26.38
rs72963110	*AC090457.1* (lincRNA)	26,531 bp	Intergenic	No	Rank = 5, Score = 0.58955	1.34

**Table 5:** SNP associated with azoospermia/severe oligozoospermia and miRNA gain/loss of binding sites according to miRNASNP-v3 database [21].

SNP	Gene	Gain	Loss
rs75614542	*FBXW2*	hsa-miR-7110-3p, hsa-miR-6873-3p, hsa-miR-6817-3p, hsa-miR-4680-5p, hsa-miR-618	hsa-miR-3675-3p

## Data Availability

The data presented in this study are available upon request from the corresponding author.

## Supplementary Materials

The following supporting information can be downloaded at: https://www.mdpi.com/article/10.3390/cimb46070389/s1, Figure S1: Quantile–quantile plot of the association analysis. The x-axis represents the expected significance level on a −log_10_ scale, while the y-axis represents the observed significance level on the same scale.
