# Supplementary figures and images for: Genetic Insights into Azoospermia and Severe Oligozoospermia: Discovering Seven SNPs through GWAS and In Silico Analysis
Source: Curr Issues Mol Biol. 2024 Jun 27;46(7):6522–32. doi: 10.3390/cimb46070389 (PMC11276099; doi:10.3390/cimb46070389)

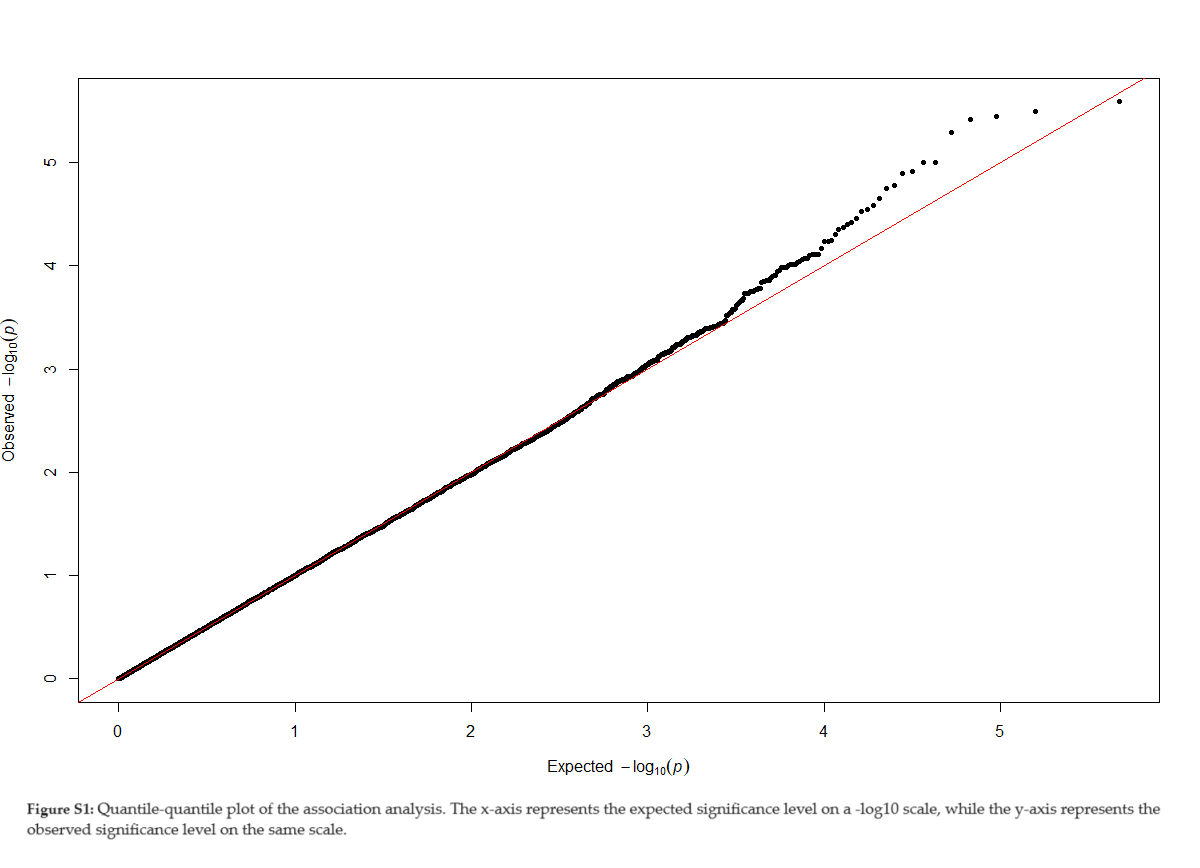

Supplement: Supplementary file 1 [file cimb-46-00389-s001.zip › Figure S1.png]
